# Mechanical Evaluation Of Unity Elevated Vacuum Suspension System

**DOI:** 10.33137/cpoj.v2i2.32941

**Published:** 2020-03-20

**Authors:** H Gholizadeh, ED Lemaire, R Salekrostam

**Affiliations:** 1 Ottawa Hospital Research Institute, Centre for Rehabilitation Research and Development, Ottawa, Canada.; 2 Faculty of Medicine, University of Ottawa, Ottawa, Canada.; 3 Faculty of Engineering, Department of Mechanical Engineering, University of Ottawa, Ottawa, Canada.

**Keywords:** Prosthesis, Amputation, Prosthetic limb, Elevated vacuum, Prosthetic suspension system, sociodemographics, Rehabilitation

## Abstract

**BACKGROUND:**

Small residual limb-socket displacement is a good indicator of prosthetic suspension system quality. Active vacuum suspension systems can decrease vertical movement inside the socket, compared to non-active suction systems. This study mechanically evaluated limb-socket displacement with the Össur Unity active vacuum system.

**METHOD:**

Forty-eight conditions were evaluated: four cylindrical and four conical sockets (polypropylene, polyethylene terephthalate glycol-modified (PETG), thermoset resin (acrylic), Thermolyn soft materials); two Iceross Seal-In V liners (standard, high profile); three vacuum conditions (active vacuum, inactive vacuum, no suction with valve open). An Instron 4428 test machine applied 0-100N linear ramped tensile loads to each positive mold, with the socket secured in place, while displacement between the mold and socket was recorded. Following the displacement tests, the load before failure (i.e., 10 mm displacement) was measured.

**RESULTS:**

Average and standard deviations for movement between the mold and sockets were small. The displacement average for all conditions was 0.30±0.16mm for active vacuum, 0.32±0.16mm for inactive vacuum, and 0.39±0.22mm for no suction. Across all trials, active vacuum systems tolerated significantly (p<0.001) more load before failure (812±221N) compared to inactive vacuum (727±213N), and no suction (401±184N). The maximum load before failure (1142±53N) was for the cylindrical polypropylene socket and high-profile liner.

**CONCLUSION:**

The Unity system successfully controlled pistoning inside the socket for regular activity loads and also controlled the greatest traction loads. While relative movement was smallest for Unity, all conditions (inactive vacuum, no suction) were viable for loads less than 100N. Furthermore, similar results can be achieved when using different socket fabrication materials.

## INTRODUCTION

The method for attaching a prosthesis to the body is termed prosthetic suspension. Selecting a suitable suspension system is an important step in the prosthetic rehabilitation process.^1–4^ A better understanding of prosthetic suspension systems may facilitate selection based on the amputee’s needs, leading to better socket system performance.^2,5,6^ Small residual limb-socket displacement is a good indicator of prosthetic suspension system quality.^7,8^

Various prosthetic suspension approaches are used in clinical practice. A thigh corset was traditionally used for suspension, but introduction of the patellar-tendon bearing prosthesis lead to other suspension methods; such as, cuff, supracondylar-suprapatellar, and suprapatellar strap.^2,6^ The Icelandic roll-on silicone socket (ICEROSS) system was introduced to the rehabilitation market to improve suspension via close adhesion of the silicone liner to the residual limb skin.^2,6^ Various methods are used to hold the silicone liner inside the prosthetic socket; including, single distal pin/lock, lanyard, suction, seal-in, or vacuum. A standard lock system for all amputees has not been defined.^1,2,6^

Vacuum assisted suspension systems (VASS) add an externally generated vacuum to a liner-based suspension system to decrease pistoning within the socket, reduce residual limb volume loss over time, and improve prosthesis control and proprioception.^1,3,9,10^ Street^11^ mentioned that vacuum could eliminate movement and reduce shear, provide a healthier environment for the residual limb, and prevent volume loss. Rosenblatt^12^ also reported that elevated vacuum systems can improve maximum walking speed, comfort, and gait symmetry compared to suction sockets and sleeve suspension systems.^12^ While elevated vacuum systems may have some benefits over the other suspension systems and improve amputee quality of life, these systems may not be appropriate for all amputees since donning the prosthesis requires more procedures, and amputees must deal with the liners, sleeves, controls, etc.^13–16^ Moreover, air between the liner and skin may create skin blisters.^16^

Recently, the Unity elevated vacuum suspension system (https://assets.ossur.com/library/31882/IFU) was developed by Össur. Unity consists of a mechanical vacuum pump in the foot shell, which uses prosthetic foot motion to draws air out from the socket in each step. Unity includes a hypobaric sealing membrane around a silicon liner so that an external sleeve is not required, unlike other vacuum systems on the market such as Harmony (Ottobock) or LimbLogicVS (Ohio Willow Wood). External sleeves can restrict knee range of motion, retain heat and therefore create perspiration problems, and may be replaced regularly due to sleeve punctures.^2^

Seal-In V liners are used and are available as standard and high profile options with only cylindrical shape. The high profile liner has a more proximal sealing membrane and is used when the person has sensitive locations on the distal tibial crest or a long residual limb. The high profile liner may have better pistoning control than standard profile due to larger vacuum area. The manufacturer suggests a thin layer of polyethylene terephthalate glycol-modified (PETG) materials over the positive cast before socket fabrication with thermoset resin (acrylic). However, PETG material is rigid and not suitable for people who want flexible inner socket. Therefore, investigating other materials for socket fabrication could be of benefit to amputees.

Pistoning measurement has been used to evaluate suspension system quality for static and dynamic conditions.^7,8,13,14,18–22^ Testing involves applying a tensile load to the socket and then measuring displacement between the socket and limb. Test loads are based on swing phase forces during gait, with typical prosthetic limb tensile loads of 44.5N during walking and 88.9N during running.^1,7^ This load is applied to the suspension system in less than one second and depends on prosthetic weight and walking speed.

Currently, research is lacking on how the Unity elevated vacuum system controls pistoning within the socket, with different socket materials. This information is important to guide prosthetic prescription and to characterize this technology within the current scope of prosthetic suspension systems. Therefore, mechanical testing was conducted to provide quantitative evidence to guide clinical practice.

## METHODOLOGY

**Figure 1:**
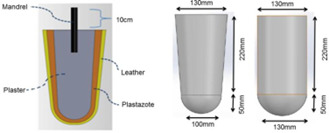
Positive mold. Left: cross-sectional view of positive mold (conical shape); Right: socket dimensions.

**Figure 2:**
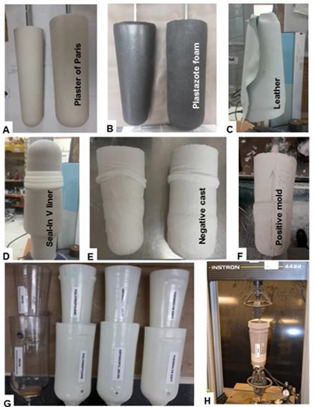
Sockets fabrication and mechanical testing. A-G: Process of making cylindrical and conical sockets; H: mechanical testing.

The Össur Unity elevated vacuum suspension system was used in this project. To provide a repeatable and standardized socket and limb surrogate, two reusable positive model (cylindrical and conical shapes) were made from plaster and covered with Plastazote medium foam (2cm) and leather to simulate skin and soft tissue (Figure 1). The model dimensions were obtained from Campbell, et al.^23^ Each positive model retained a steel mandrel in the proximal end. The reusable positive molds were only used during mechanical testing and not for socket fabrication. To avoid Plastazote compression under vacuum while laminating/thermoforming the sockets (Figure 2), molds were casted with Plaster of Paris (Figure 2, E) and then reusable positive plaster models were fabricated (Figure 2, F). The positive models were used for socket fabrication.

The cylindrical and conical mold were used to fabricate eight sockets, based on the Unity manufacturer guidelines, with standard and high profile Iceross Seal-In V liners and four materials: polypropylene (PP), polyethylene terephthalate glycol-modified (PETG), thermoset resin (acrylic), Thermolyn soft (Figure 2). A custom adaptor was attached to the socket’s distal end. A valve from the Unity transtibial kit was attached to the socket wall below the liner seal position.

Socket displacement was measured for active vacuum (-18inHg), inactive vacuum (i.e., acting as a suction suspension system), and no suction (i.e., valve in open position) conditions. As shown in Figure 2(H), the prosthetic foot vacuum pump was attached to the socket and a pressure gauge (Mini Dial Air Pressure Gauge Meter) was used to check negative pressure inside the socket. The gauge was in series with the UNITY tube and pump. We created -18inHg for active vacuum by simulating prosthetic foot motion to draw air out from the socket. An Instron 4482 tensile test machine was used to apply ramped tensile loads and measure displacement. The positive mold’s mandrel was attached to the Instron’s superior grip and the socket’s distal adapter was connected to the Instron’s inferior grip (i.e., fixed, non-moving attachment). The positive molds were pulled with linear ramped loads from 0 to 100 N, over 1 second. A 100N maximum load encompasses the typical range of lower limb loads in daily living.^7,21^ Following the displacement tests, the load that each condition can tolerate before the suspension failed was measured. A 10-mm displacement between mold and socket was considered as failure since amputees consider sockets to fit well and to be secure with displacements of less than 10 mm.^8^

**Figure 3:**
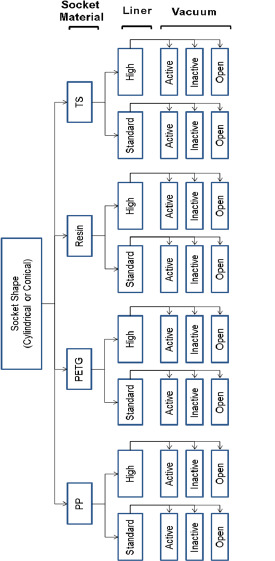
Tensile tests (48 combinations). PP (polypropylene); PETG (polyethylene terephthalate glycol-modified); Resin (thermoset resin); TS (Thermolyn soft).

A total of 48 test conditions were evaluated (Figure 3). These conditions included: socket shape (cylindrical, conical), socket material (polypropylene, polyethylene terephthalate glycol-modified, thermoset resin, Thermolyn soft material), liner seal position (high profile, standard profile), and vacuum (active vacuum, inactive vacuum, no suction). Ten trials were completed for each test condition. A review paper by Eshraghi et al.,^7^ showed that different techniques have been used to measure pistoning inside the socket and only five studies^21,24–27^ completed 3 to 5 trials due to ethical considerations related to the x-ray exposure. This study included 10 trials which provide more reliable analysis than other reviewed studies. The liners and mold were examined after each test to ensure that no damage or changes occurred. Moreover, a minimum of 5 minutes between tests allowed the Plastazote to return to its original shape.

### Data Analysis

Positive mold displacements were extracted using the Instron Bluehill 2 Software and imported into Excel for analysis. Maximum displacement was determined for each 100N load trial and averages and standard deviations (SD) were calculated across the 10 trials for each test condition. For the suspension failure tests, the failure load was determined for each trial and averages and SD were calculated across the 10 trials for each test condition. Statistical analyses were performed using SPSS 23 and the normality of variables was verified by the Shapiro-Wilk Normality test. A one-way Repeated Measures Analysis of Variance (ANOVA) with post hoc Bonferroni analysis was used to compare active vacuum, inactive vacuum, and no suction conditions. Moreover, a paired samples t-test was used to compare Seal-In V high profile and standard profile liners. The significance level was set at 0.05.

## RESULTS

Average and standard deviations for movement between the positive molds and sockets were small (Table 1). Across all conditions, the average displacement was 0.30±0.16mm for active vacuum, 0.32±0.16mm for inactive vacuum, and 0.39±0.22mm for no suction. Overall, less movement was measured with conical socket shapes (0.28±0.16mm) compared to cylindrical sockets (0.33±0.17mm) in active vacuum condition when 100N traction load applied.

**Table 1:** Average and standard deviations for displacement (mm) and load (N) before failure.

**Socket**	**Liner**	**Vacuum**	**Displacement at 100N load**	p-value*	**Load at 10mm displacement**	p-value*
**Resin conical**	Standard profile	ON	0.45±0.04	(1,2) 1.000	562.4±14.10	(1,2) 0.001
		OFF	0.45±0.04	(1,3) 1.000	521.3±5.22	(1,3) 0.001
		Valve open	0.47±0.05	(2,3) 1.000	214.2±12.78	(2,3) 0.001
	High profile	ON	0.22±0.03	(1,2) 1.000	701.5±10.85	(1,2) 0.001
		OFF	0.22±0.01	(1,3) 0.001	562.6±15.96	(1,3) 0.001
		Valve open	0.30±0.02	(2,3) 0.001	258.0±5.60	(2,3) 0.001
**Resin cylindrical**	Standard profile	ON	0.37±0.01	(1,2) 0.237	711.9±4.80	(1,2) 0.001
		OFF	0.38±0.02	(1,3) 0.001	671.1±3.57	(1,3) 0.001
		Valve open	0.61±0.03	(2,3) 0.001	252.1±2.35	(2,3) 0.001
	High profile	ON	0.24±0.04	(1,2) 0.146	905.7±28.02	(1,2) 0.001
		OFF	0.27±0.01	(1,3) 0.206	706.7±31.72	(1,3) 0.001
		Valve open	0.27±0.01	(2,3) 1.000	476.7±8.81	(2,3) 0.001
**PETG conical**	Standard profile	ON	0.60±0.11	(1,2) 1.000	442.2±42.37	(1,2) 0.026
		OFF	0.60±0.07	(1,3) 0.022	398.5±45.00	(1,3) 0.001
		Valve open	0.76±0.10	(2,3) 0.010	151.34±14.70	(2,3) 0.001
	High profile	ON	0.19±0.01	(1,2) 0.222	786.5±55.11	(1,2) 0.986
		OFF	0.20±0.02	(1,3) 0.001	764.5±32.66	(1,3) 0.001
		Valve open	0.21±0.01	(2,3) 0.019	453.7±14.37	(2,3) 0.001
**PETG cylindrical**	Standard profile	ON	0.71±0.23	(1,2) 1.000	741.9±19.32	(1,2) 0.037
		OFF	0.74±0.08	(1,3) 0.314	711.4±22.48	(1,3) 0.001
		Valve open	0.92±0.10	(2,3) 0.002	323.5±10.53	(2,3) 0.001
	High profile	ON	0.20±0.02	(1,2) 0.153	1060.5±20.48	(1,2) 0.076
		OFF	0.22±0.06	(1,3) 0.001	915.8±140.81	(1,3) 0.001
		Valve open	0.24±0.01	(2,3) 0.813	663.7±116.49	(2,3) 0.013
**Polypropylene conical**	Standard profile	ON	0.23±0.10	(1,2) 0.413	446.6±14.98	(1,2) 0.001
		OFF	0.29±0.02	(1,3) 0.439	324.8±5.02	(1,3) 0.001
		Valve open	0.30±0.06	(2,3) 1.000	280.3±31.16	(2,3) 0.001
	High profile	ON	0.14±0.02	(1,2) 0.020	805.3±18.35	(1,2) 0.001
		OFF	0.19±0.01	(1,3) 0.001	701.4±31.07	(1,3) 0.001
		Valve open	0.23±0.01	(2,3) 0.001	323.1±17.75	(2,3) 0.001
**Polypropylene cylindrical**	Standard profile	ON	0.38±0.10	(1,2) 0.657	751.9±54.63	(1,2) 0.001
		OFF	0.38±0.03	(1,3) 0.001	688.5±11.19	(1,3) 0.001
		Valve open	0.54±0.02	(2,3) 0.001	277.1±1.69	(2,3) 0.001
	High profile	ON	0.17±0.03	(1,2) 0.141	1141.7±52.64	(1,2) 0.003
		OFF	0.19±0.01	(1,3) 0.032	1061.7±40.78	(1,3) 0.001
		Valve open	0.20±0.01	(2,3) 0.158	709.0±99.65	(2,3) 0.001
**Thermolyn soft conical**	Standard profile	ON	0.20±0.01	(1,2) 1.000	855.2±23.82	(1,2) 0.002
		OFF	0.20±0.01	(1,3) 0.496	801.3±29.39	(1,3) 0.001
		Valve open	0.21±0.01	(2,3) 0.261	469.6±11.39	(2,3) 0.001
	High profile	ON	0.18±0.01	(1,2) 0.211	1126.4±36.97	(1,2) 0.001
		OFF	0.19±0.01	(1,3) 0.001	1073.2±11.26	(1,3) 0.001
		Valve open	0.20±0.01	(2,3) 0.004	748.4±28.84	(2,3) 0.001
**Thermolyn soft cylindrical**	Standard profile	ON	0.33±0.02	(1,2) 0.732	793.0±40.24	(1,2) 0.001
		OFF	0.34±0.02	(1,3) 0.001	752.2±14.14	(1,3) 0.001
		Valve open	0.47±0.03	(2,3) 0.001	285.9±10.38	(2,3) 0.001
	High profile	ON	0.21±0.04	(1,2) 1.000	1136.1±58.83	(1,2) 0.001
		OFF	0.21±0.01	(1,3) 0.060	971.0±13.56	(1,3) 0.001
		Valve open	0.23±0.01	(2,3) 0.009	528.6±10.92	(2,3) 0.001

* P values for valve setting comparisons: 1=ON, 2=OFF, 3=valve open.

Overall, active vacuum systems tolerated significantly (p<0.001) more load before failure (812±221N), compared to inactive vacuum (727±213N) and no suction (401±184N). With active vacuum, the maximum load before failure (1142±53N) was for the cylindrical polypropylene socket and high-profile liner. The minimum load was recorded with the PETG conical socket and standard profile liner in active vacuum (442±42N) and open valve conditions (151± 15N). For the passive condition, the minimum load was recorded with the Polypropylene conical socket and standard profile liner (324.8±5N).

**Figure 4:**
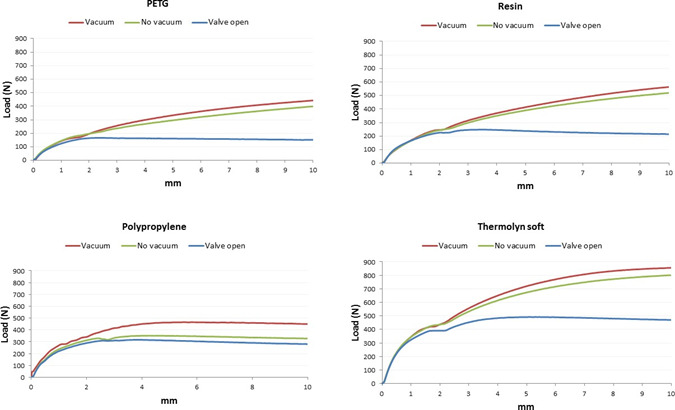
Average displacement (mm) and load (N) in conical socket with Seal-In V standard profile.

With active vacuum, an average of 958±179N was required to displace positive molds with high profile liners, compared to 665±155N for standard liners (p<0.001). Moreover, the two socket shapes were significantly different (p<0.01) since more load was needed for 10 mm displacement in cylindrical sockets (905±183N), compared to conical sockets (718±227N) when using active vacuum (Table 1, Figure 4-Figure 7).

**Figure 5:**
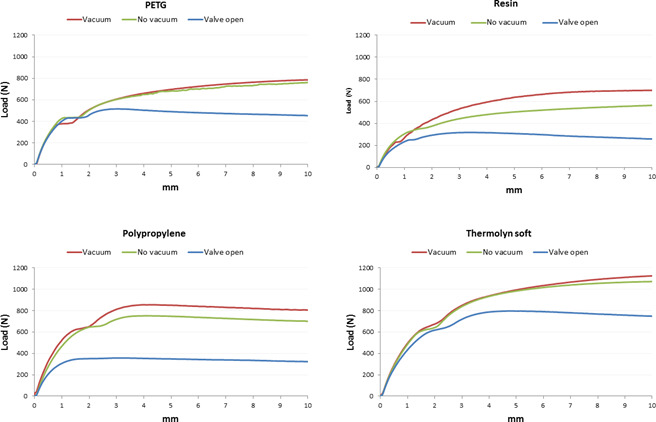
Average displacement (mm) and load (N) in conical socket with Seal-In V high profile.

**Figure 6:**
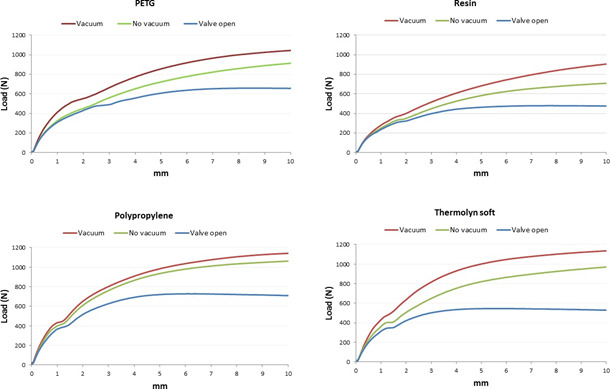
Average displacement (mm) and load (N) in cylindrical socket with Seal-In V high profile.

**Figure 7:**
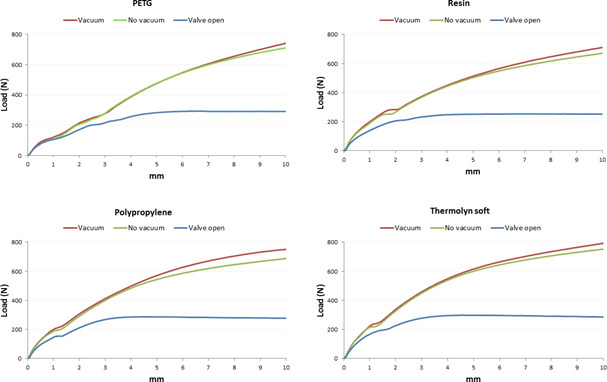
Average displacement (mm) and load (N) in cylindrical socket with Seal-In V standard profile.

## DISCUSSION

Mechanical testing was conducted to evaluate the Össur Unity elevated vacuum suspension system’s ability to minimize socket displacement when external traction forces are applied. The results of this study showed that the Unity system successfully controlled socket pistoning and different materials can be used for socket fabrication.

From the literature, vacuum and suction suspension systems could diminish residual limb displacement inside the socket.^13,28^ Klute et al. measured pistoning with a motion analysis system while their participants stood in place and shifted their weight from side to side (i.e., weighted and un-weighted).^13^

Residual limb pistoning was significantly less (1±3mm) with the Harmony suspension system providing active vacuum than a pin/lock system (6±4mm).^13^ The results of this study showed that after applying 100N traction load, only 0.30±0.16 mm of displacement was found with active vacuum across all conditions.

Another study^21^ revealed that adding up to 90N loads to the prosthesis caused less pistoning in a suction socket using Seal-In X5 liner (2±1mm) compared to a pin/lock system (5±2 mm). In the current study, 0.32±0.16mm pistoning inside the suction socket was recorded overall (average for all conditions). This result is lower than the previous studies on different suspension system^13,21^ and showed that the Seal-In V could control the movement inside the socket successfully.

Gholizadeh et al., 2014, showed that a Seal-In X5 liner with PETG socket tolerated 310N loads before suspension failure, pin/lock systems tolerated 580 N, and magnetic suspension systems tolerated 351N.^29^ In this study, the maximum load that each condition could tolerate before suspension failure was up to 86% higher than other studies in the literature.^29,30^ While the manufacturer suggested PETG materials for the Unity socket fabrication, the results of this study showed that different materials can be used without sacrificing suspension performance.

Wirta et. al.,^31^ compared the vertical movement of conical and cylindrical residual limb shapes with patellar tendon bearing (PTB) sockets and different suspension systems (i.e., supracondylar/suprapatellar, supracondylar, cuff, waistband and cuff, figure-eight strap, rubber sleeve, articulated supracondylar wedge). In both conical and cylindrical residual limbs, the rubber sleeve produced the least pistoning of the seven evaluated systems (ranged from 6 to 31mm for all system), and the cylindrical residual limb had more pistoning compared to conical stump.^31^ Similarly, less movement was seen in this study with conical socket shape compared to cylindrical socket when 100N traction load applied, but the average difference was small (0.05 mm).

Literature has shown that donning and doffing the prosthesis is challenging for elderly amputees using Seal-In X5 liner.^2,22^ This study showed that a minimum of 151±15N was needed with the PETG conical socket and standard profile liner in open valve condition to pull off the socket, and a maximum of 748±291N was needed for the Thermolyne soft socket. Therefore, PETG would be good choice for amputees who may have difficulty doffing their prosthesis.

### Limitations

In this study, plaster positive molds were used and covered with Plastazote and leather,^32^ and were pulled straight using an Instron test machine. In practice, the prosthesis user could wiggle their residual limb to remove their prosthesis with less force. Future research is needed to evaluate donning/doffing procedures in transtibial amputees. Reusable molds, covered with Plastazote foam and leather, were used to represent a residual limb; however, silicone materials might be a better option to simulate the soft tissue than Plastazote.^33^

## CONCLUSION

The Unity system successfully controlled pistoning inside the socket for regular activity loads and also controlled the greatest traction loads. While relative movement was smallest for the Unity, the inactive vacuum (suction) condition was also viable for loads less than 100N. This study showed that the Unity system can hold the residual limb inside the socket successfully even if there is a failure in the vacuum pump.

## DECLARATION OF CONFLICTING INTERESTS

The authors have declared that no competing interests exist. No commercial party having a direct financial interest in the results of the research supporting this article has or will confer a benefit on the authors or on any organization with which the authors are associated.

## AUTHOR CONTRIBUTION

- **Hossein Gholizadeh**,
  designed the system and the protocol, conducted the experiments, collected and analyzed the data, discussed the results and drafted the manuscript.
- **Edward D Lemaire**,
  supervised the overall project, and helped in writing and revising the manuscript.
- **Rasool Salekrostam**,
  conducted mechanical testing, collected and analyzed the data.

## SOURCES OF SUPPORT

This study was financially supported by Össur and Mitacs.
